# Supplementary figures and images for: Genome-Wide Identification and Characterization of Long Non-Coding RNAs in Peanut
Source: Genes (Basel). 2019 Jul 15;10(7):536. doi: 10.3390/genes10070536 (PMC6679159; doi:10.3390/genes10070536)

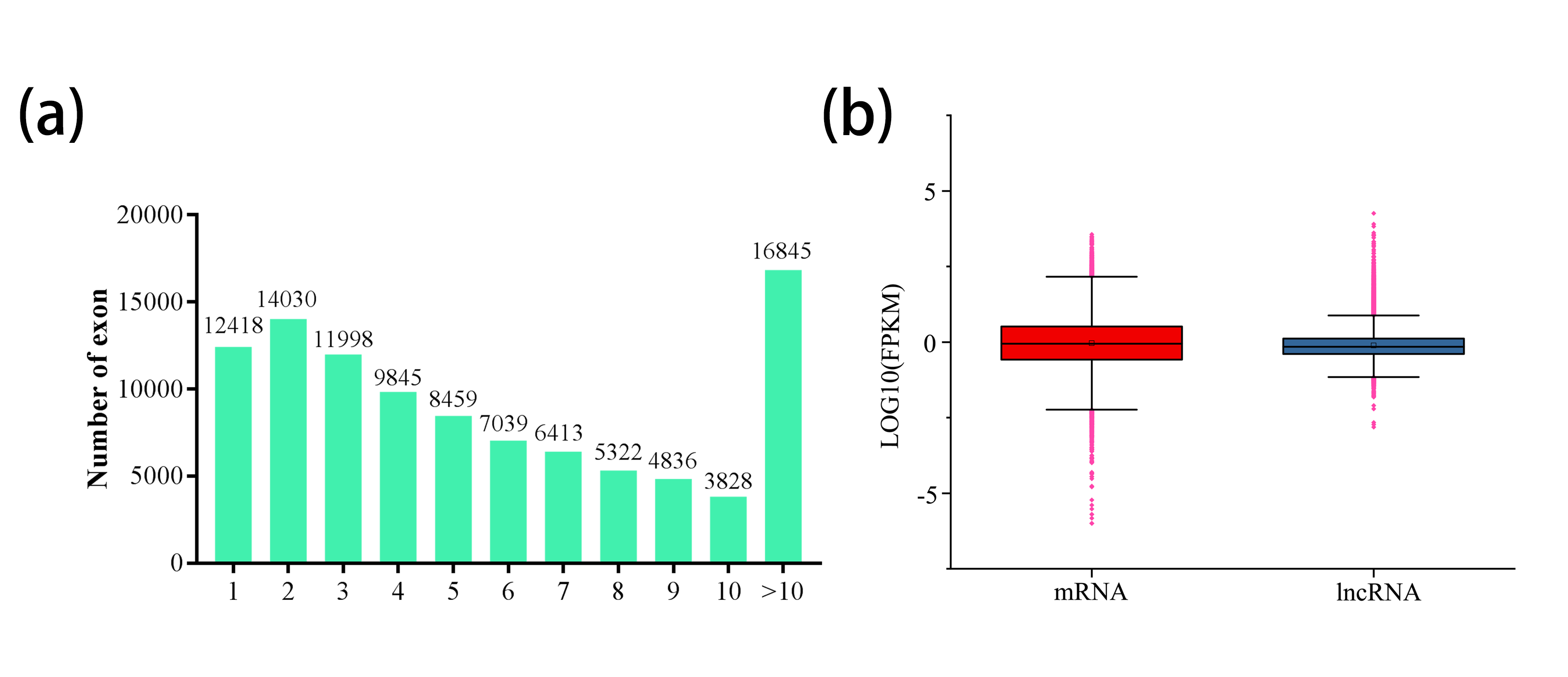

Supplement: Supplementary file 1 [file genes-10-00536-s001.zip › Supplementary Files/Figure S1.tif]

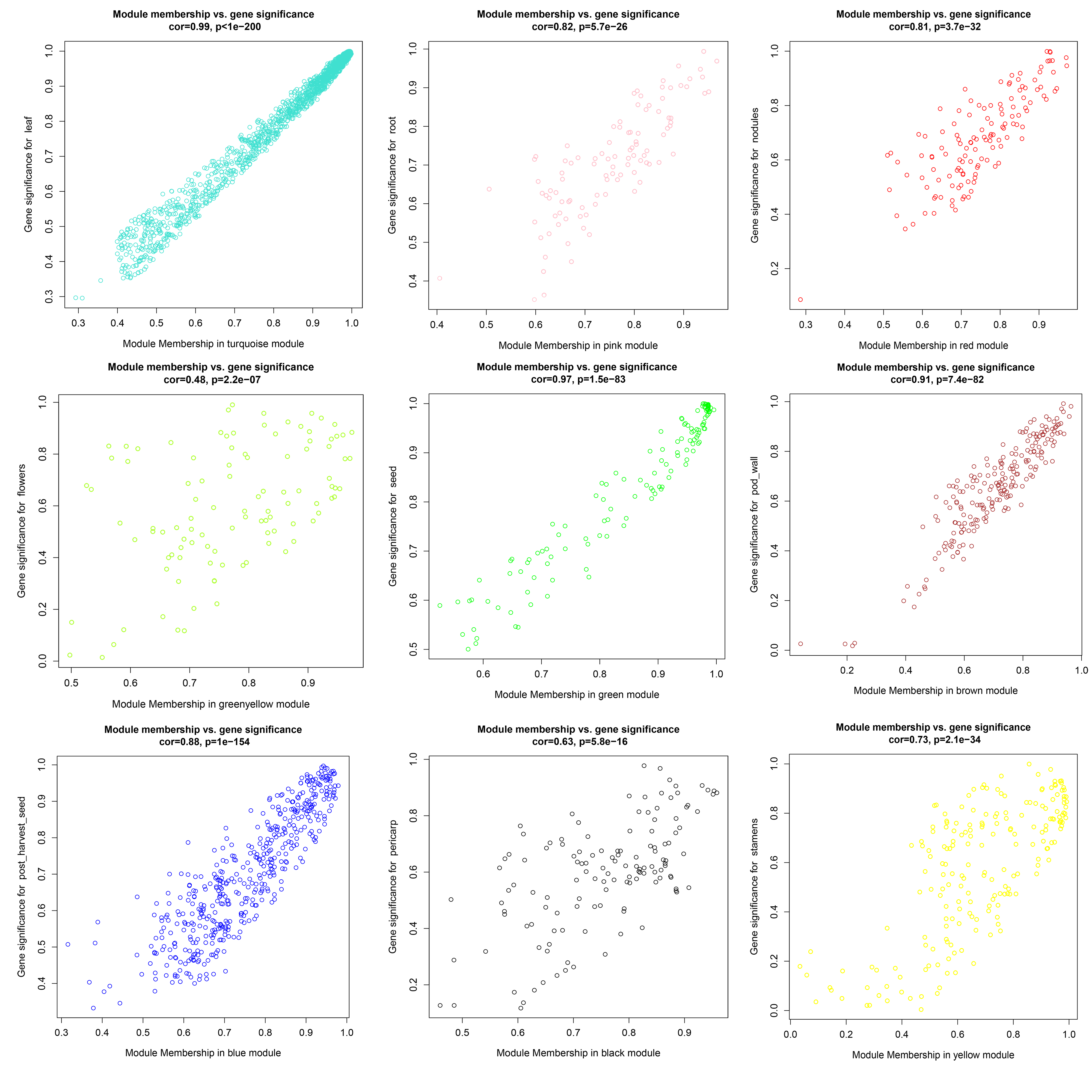

Supplement: Supplementary file 1 [file genes-10-00536-s001.zip › Supplementary Files/Figure S2.tif]

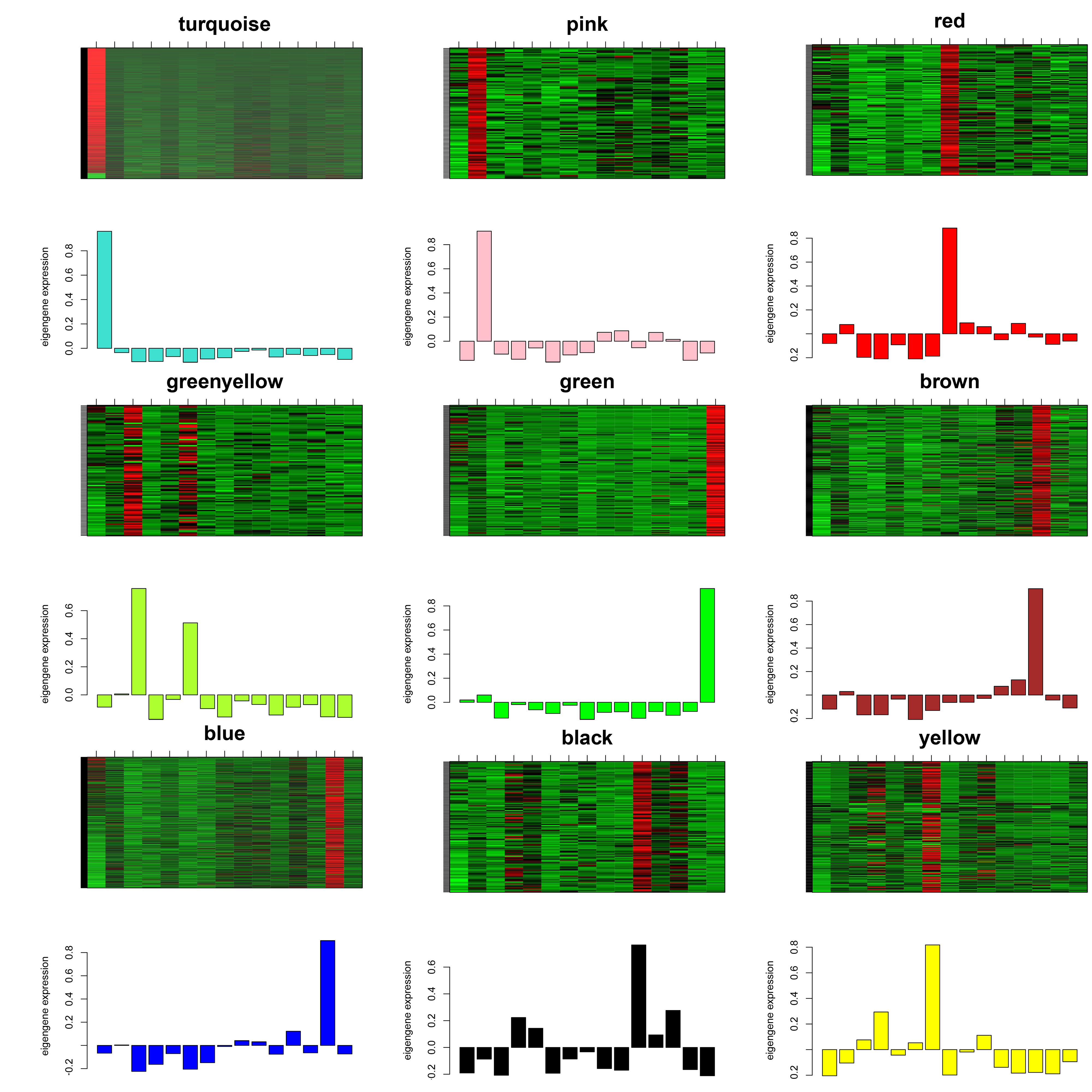

Supplement: Supplementary file 1 [file genes-10-00536-s001.zip › Supplementary Files/Figure S3.tif]

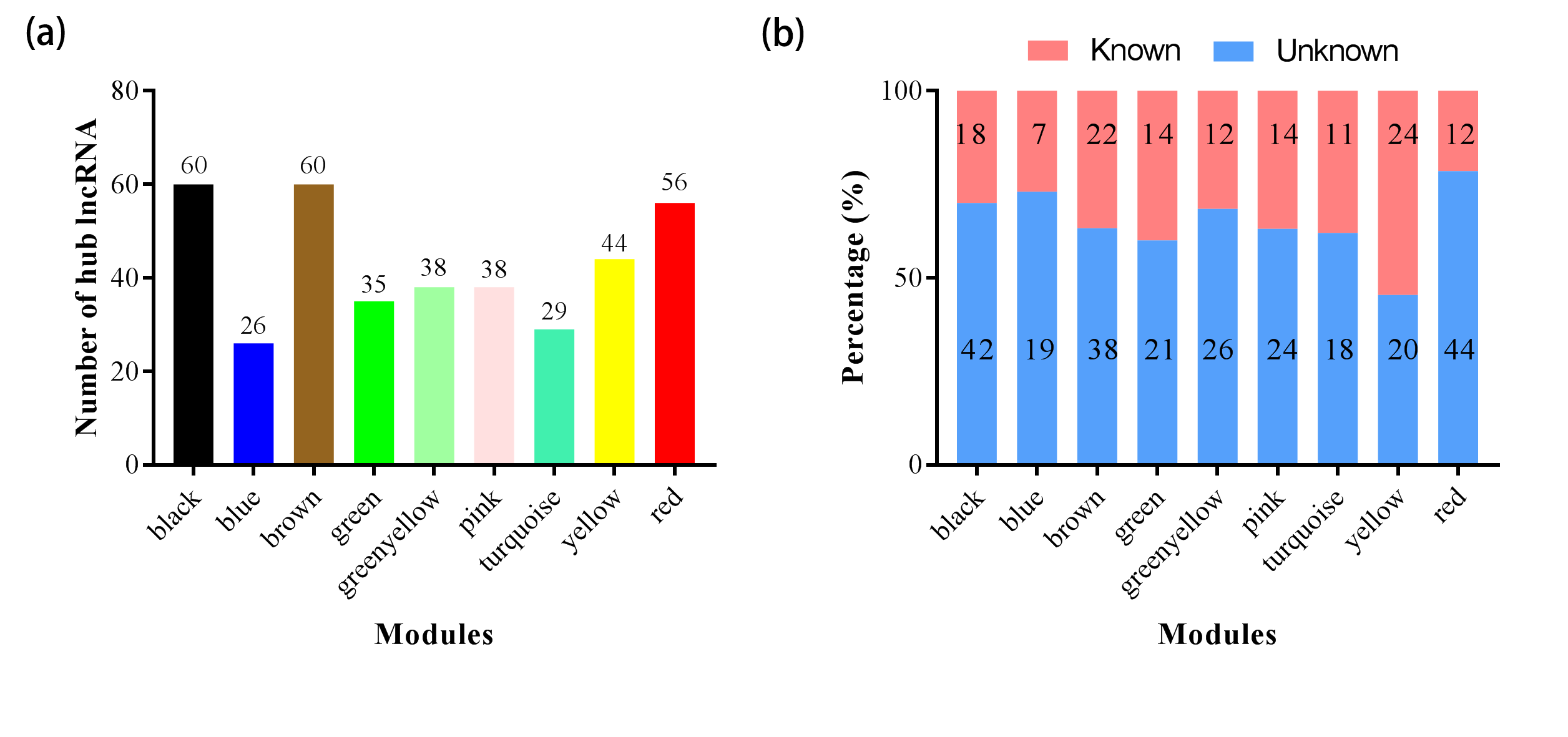

Supplement: Supplementary file 1 [file genes-10-00536-s001.zip › Supplementary Files/Figure S4.tif]
